# A dataset of radar-recorded heart sounds and vital signs including synchronised reference sensor signals

**DOI:** 10.1038/s41597-020-0390-1

**Published:** 2020-02-13

**Authors:** Kilin Shi, Sven Schellenberger, Christoph Will, Tobias Steigleder, Fabian Michler, Jonas Fuchs, Robert Weigel, Christoph Ostgathe, Alexander Koelpin

**Affiliations:** 1 Institute for Electronics Engineering, Friedrich-Alexander-Universität Erlangen-Nürnberg (FAU), 91058 Erlangen, Germany; 2 Chair of Electronics and Sensor Systems, Brandenburg University of Technology, 03046 Cottbus, Germany; 3 Department of Palliative Medicine, Universitätsklinikum Erlangen, Comprehensive Cancer Center CCC Erlangen - EMN, Friedrich-Alexander-Universität Erlangen-Nürnberg (FAU), 91054 Erlangen, Germany

**Keywords:** Biomedical engineering, Preclinical research, Data acquisition

## Abstract

Radar systems allow for contactless measurements of vital signs such as heart sounds, the pulse signal, and respiration. This approach is able to tackle crucial disadvantages of state-of-the-art monitoring devices such as the need for permanent wiring and skin contact. Potential applications include the employment in a hospital environment but also in home care or passenger vehicles. This dataset consists of synchronised data which are acquired using a Six-Port-based radar system operating at 24 GHz, a digital stethoscope, an ECG, and a respiration sensor. 11 test subjects were measured in different defined scenarios and at several measurement positions such as at the carotid, the back, and several frontal positions on the thorax. Overall, around 223 minutes of data were acquired at scenarios such as breath-holding, post-exercise measurements, and while speaking. The presented dataset contains reference-labeled ECG signals and can therefore easily be used to either test algorithms for monitoring the heart rate, but also to gain insights about characteristic effects of radar-based vital sign monitoring.

## Abstract (summary)

**Table:** 

Measurement(s)	heart function measurement • heart rate • Respiration • heart sounds
Technology Type(s)	radar system
Factor Type(s)	sex • age • weight • height • BMI
Sample Characteristic - Organism	Homo sapiens

Machine-accessible metadata file describing the reported data: 10.6084/m9.figshare.11778900

## Background & Summary

Vital signs, such as heart rate and respiratory rate, are key parameters when assessing the physical condition of a person. State-of-the-art technologies for vital sign monitoring include the electrocardiograph (ECG), the photoplethoysmograph (PPG), or the phonocardiograph (PCG). While the ECG makes use of the electrical stimulation that triggers each heartbeat^1^, the PPG measures the changing absorbance caused by the blood flow^2^ and the PCG measures the acoustic waves that result from the mechanical activity of the heart. All current devices have the common disadvantage that they need to be in permanent contact with the person. Apart from severely restricting the independence and mobility^3,4^, irritation by the electrodes might lead to undue distress and even increase the symptom burden^5^. Furthermore, the manipulation of the probes might lead to false alarms, which in turn leads to a decreased responsiveness of medical personnel to automated alarms, a phenomenon which is called alarm fatigue^6^. Nonetheless, continuous monitoring is essential to detect anomalies and diseases. A more convenient technology is ballistocardiography^7,8^, which however is restricted to bed-ridden persons and standardized conditions. Further options of continuous monitoring comprise more complex devices such as infrared camera-based systems, which however requires several markers on the person^9^, and distance-measuring devices such as laser-^10–12^ or radar-based^13–26^ systems. When comparing the latter two, radar systems have the advantage that they are able to penetrate clothing and allow for a very comfortable way of continuous and contactless vital sign monitoring. Using contactless distance measurements, vibrations of the thorax can be recorded in order to reconstruct vital parameters such as respiration, the pulse signal, and even the heart sound signal. Park *et al*.^26^ evaluated a ultra-wideband radar system for respiration and carotid pulse monitoring. Besides healthy test subjects with normal sinus rhythm they also included subjects with persistent atrial fibrillation. For all test subjects, they show that the radar system was able to reliably measure the respiration, carotid pulse, and heart rhythm. Mercuri *et al*.^23^ use a radar system with one transmitting and two receiving antennas to monitor vital signs of multiple people. They are able to track targets and reject random body movements in order to obtain the vital signs of individual persons. Other researchers, such as Liang *et al*.^25^ have presented approaches to detect human vital signs behind obstacles such as a wall. These systems could be potentially used for search and rescue or indoor positioning tasks. Will *et al*. used a Six-Port-based system to prove the measurability of heart sounds using radar. These signals, that are usually measured using a stethoscope, can be analyzed to determine the heart rate but also potential diseases such as valve insufficiency or stenosis^27,28^. Li *et al*.^14,15^ and Pisa *et al*.^19^ have given overviews over some recent advances in the field of radar vital sign monitoring.

Although there has been more and more research on this topic, no public database has been released so far. A common database of radar-recorded vital signs would, e.g., allow for a comparison of algorithms that extract parameters such as the heart rate or respiration rate. Until now, there have been no standardized methods or devices for recording data that would facilitate an objective comparison. In order to create such as database, radar vital sign signals from 11 test subjects have been recorded employing standardized procedures and methods. Synchronously, reference sensor signals such as an ECG signal, a respiration sensor signal, and a PCG signal as a reference for the heart sound component within the radar signal have been measured. In our previous work, this data has partially been used to demonstrate the ability of radar systems to measure heart sounds^17^. The proposed database would allow for analyzing further questions such as the analysis of the respiration signals or the impact of changes in the scenario, such as speech, movement, or physical activity on the radar signals. The whole database consists of 13 376 s of synchronous radar, ECG, PCG, and respiration sensor data. For the radar, a Six-Port-based continuous wave radar operating at 24.17 GHz is used to comply with the industrial, scientific and medical (ISM) radio band. A three-lead ECG and an electronic stethoscope serve as reference sensors for cardiovascular activity while a temperature-based airflow sensor is used as a reference for respiration.

## Methods

### Participants

Approval was acquired from the local ethics committee before planning the experiments. Seven male and four female test subjects where measured with an average age of 34.73 ± 15.94 years and an average BMI of 23.19 ± 3.61 kg/m^2^. All test subjects were healthy and were briefed about the experiments that were conducted. A written consent was obtained from all participants that also allows for sharing the anonymized data. After briefing, the participants were asked to fill out a measurement protocol which includes their personal data such as age, sex, weight, and height. A summary of all participants is displayed in Table 1.

**Table 1:** Overview of all test subjects.

PUT^a^	Age	Sex^b^	Height (cm)	Weight (kg)	BMI^c^
1	24	M	189	100	28,0
2	26	M	183	77	23,0
3	26	M	182	58	17,5
4	52	M	183	86	25,7
5	55	M	176	78	25,2
6	25	F	167	54	19,4
7	26	M	180	76	23,5
8	69	F	167	78	28,0
9	24	F	173	60	20,0
10	27	F	163	52	19,6
11	28	M	178	80	25,2
Mean ± SD^d^	34.73 ± 15.94	—	176.45 ± 8.13	72.64 ± 14.89	23.19 ± 3.61

^a^Person-under-test. ^b^M: male, F: female. ^c^Body mass index. ^d^Standard deviation.

### Human subjects

The study was approved by the ethics committee of the Friedrich-Alexander-Universität Erlangen-Nürnberg (No. 85_15B). All research was performed in accordance with relevant guidelines and regulations. The informed consent was obtained from all subjects in human trials.

### Procedures

**Fig. 1:**
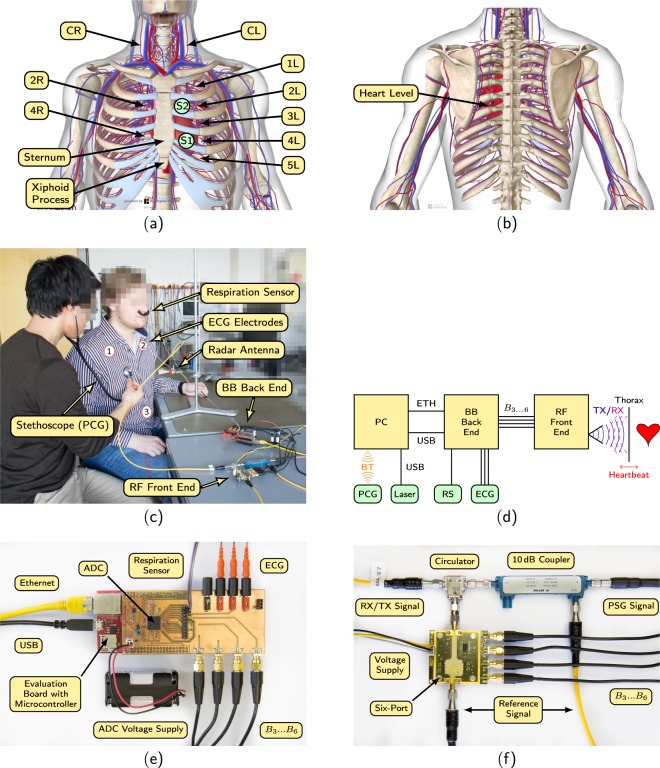
Overview of the different measurement spots, the measurement setup, and the system configuration. (**a**) Measurement spots at the thorax^17^. (**b**) Measurement spot at the back. (**c**) Experimental setup with a test subject in a sitting position. The informed consent was obtained from the persons in the image. (**d**) Block diagram of the overall setup^17^. (**e**) Photograph of the BB back end^17^. (**f**) Photograph of the RF front end^17^. Images in (**a)** and (**b**) are taken from Biodigital Inc. (https://human.biodigital.com/index.html).

All measurements were recorded at the Institute for Electronic Engineering at the Friedrich-Alexander-Universität Erlangen-Nürnberg. At least two supervising persons monitored the measurements and ensured a trouble-free progress of the measurements. At the beginning of each measurement, the ECG electrodes and the respiration sensor were attached to the test subject. Next, the thorax of the test subject was auscultated to locate the positions at which a strong heart sound signal could be perceived. Since the PCG served as a reference sensor, it was usually placed on a location at which a very high signal quality was observed. The antenna of the radar system had therefore to be focused on different regions at which, however, a good signal quality was still to be expected. The distance between the antenna and the region of interest (ROI) was around 20 cm during all measurements. To maximize the signal quality, the antenna direction was chosen perpendicular to the thorax surface. The length of each measurement was around 60 s. The different measurement positions on the thorax, on the back, and on the carotid of the test subjects are depicted in Fig. 1a,b. The right and left carotid are abbreviated using the terms “CR” and “CL”, respectively. The number at the other positions describes the number of the intercostal spaces starting from the top. “R” and “L” describe the position on the right or left side. Figure 1c shows an exemplary measurement in the laboratory. A block diagram of the overall setup can be seen in Fig. 1d. In the following, all components will be described in detail.

### Baseband back end

Figure 1e shows the baseband board that was used to digitize the sensor signals. The ECG, the radar signals, and the respiration sensor are attached to this back end to allow for simultaneous sampling of the signals. The signals are digitized using the 24 bit analog-to-digital converter *ADS1298* from *Texas Instruments* at a sampling rate of 2000 Hz. After conversion, the raw signals are sent to the PC via Ethernet.

### RF front end and Six-Port radar

A Six-Port is utilized as a quadrature interferometer for the radar application. A detailed description of the radar system is given in^17^. The Six-Port is a completely passive structure which basically consists of three quadrature hybrid couplers and one Wilkinson divider^29^. As indicated by its name, the Six-Port has two input and four output signals. The two input signals consist of a reference signal at a defined frequency and the signal that is received after reflection at the target. Inside the structure, the two input signals are superimposed under four relative and static phase shifts of 0°, 90°, 180°, and 270°. These signals are then down-converted using diode power detectors. The four resulting baseband signals ${B}_{3\ldots 6}$ form two differential and orthogonal signals *I* and *Q* which can be expressed as a complex number *Z*. A relative distance change of a target in front of the antenna results in a phase shift Δ$\varphi$^29^:

1 $$\Delta \varphi ={\rm{a}}{\rm{r}}{\rm{g}}\{\underline{Z}\}={\rm{a}}{\rm{r}}{\rm{g}}({B}_{5}-{B}_{6})+j({B}_{3}-{B}_{4}).$$

The relative distance change Δ*x* can be easily reconstructed from Δ$\varphi$ using^29^:

2 $$\Delta x=\frac{\Delta \varphi }{2\pi }\cdot \frac{\lambda }{2},$$

with *λ* being the known wavelength of the signal. However, since the unambiguousness range is limited, phase unwrapping has to be performed additionally. The whole RF front end can be seen in Fig. 1f. A reference signal at 24.17 GHz is generated using the *PSG Analog Signal Generator E8257D* from *Keysight*. The signal is split by a 10 dB coupler whereas the main part is fed to the antenna and the lesser part is directly fed into the Six-Port receiver^29^.

### Reference sensors

In the following, the reference sensors that are used for validation are described. They consist of an ECG, a PCG, and a respiration sensor.

#### ECG

A three channel ECG serves as main reference sensor for all cardiovascular signals. Three leads are attached to the body according to clinical standard^30^: one electrode at the right arm (RA), one at the left arm (LA), and one at the left leg (LL). The positions are indicated by the numbers 1–3 in Fig. 1c. Standard snap electrodes were used for the measurements wherein for hygiene reasons a new set of electrodes was used for each test subject. The ECG leads 3 (LA-LL) and 2 (RA-LL) according to Einthoven^30^ are recorded. Lead 1 can be simply calculated by subtracting lead 3 from lead 2.

#### PCG

A digital PCG was used as reference sensor for the heart sound signals. For this purpose, the *Electronic Stethoscope Model 3200* from *Littmann* was utilized. The PCG is connected to the PC via Bluetooth. The raw measurements are exported as .wav files and imported into *MATLAB*. After re-sampling and synchronisation, the PCG signals are also stored as an array in the .mat files. According to the documentation of the PCG, the signals are amplified between 20 … 1000 Hz and lower frequency sounds are emphasized between 20 … 200 Hz.

#### Respiration sensor

As a reference sensor for respiration, a passive temperature-based airflow sensor was employed. The air heats up quickly in the lungs when breathing due to the large surface area of the capillaries, so an increase in temperature can be registered at the nose when exhaling. Analogously, the sensor cools down when inhaling. Using this mechanism, a qualitative respiration curve can be created.

### Measurement protocol

During the measurements, various scenarios were carried out. These scenarios are described in the following. Please note that not all scenarios could be carried out with all test subjects, e.g., some could not perform the task that was needed for the post-exercise measurement. During all measurements, the participants were asked to stay calm and avoid any artifacts that might interfere with the measurements.

#### Default scenario

**Fig. 2:**
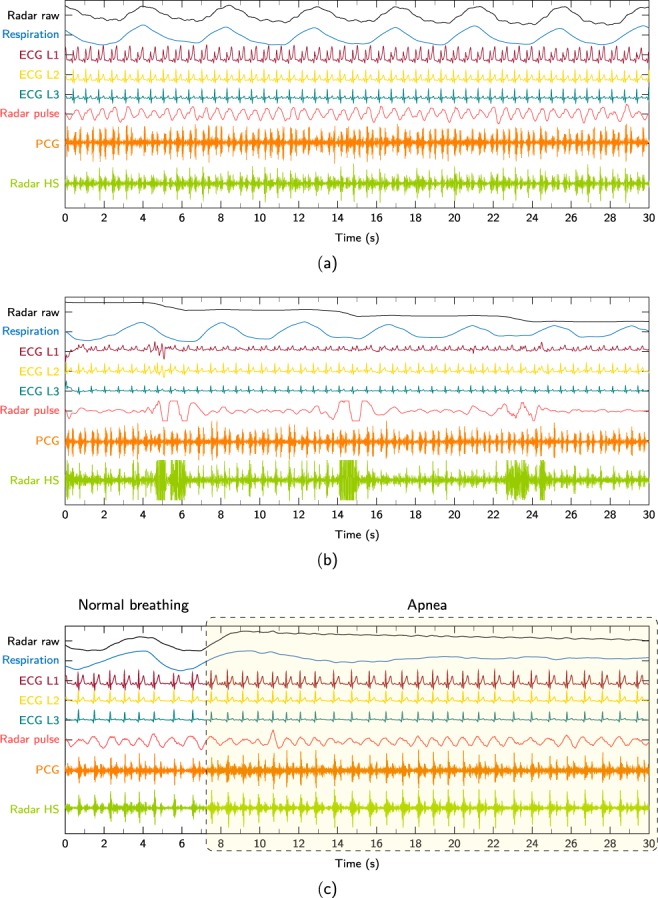
Exemplary signals of different scenarios. (**a**) Default, (**b**) Distance variation, and (**c**) Apnea scenario.

The default scenario makes up the majority of the measurements in the database. In this standard setting the test subject is sitting or standing comfortably and breathing freely. Both radar and PCG are placed on ROIs at which the heart sounds have a high signal quality. Different ROIs are possible for this scenario, however, they are all located on the thorax as seen in Fig. 1a. An exemplary illustration of a default measurement can be seen in Fig. 2a. Depicted is a segment of synchronised signals from all sensors. “Respiration”, “Radar pulse”, and “Radar HS” (heart sounds) result from filtering the “Radar raw” signal in the corresponding frequency bands.

#### Carotid

In this scenario the sensor is placed or focused on the carotid artery, either at the left or right side as seen in Fig. 1a. Heart sounds are expected to be detected at these ROIs as they travel as transverse vibrations along the ventricular walls and along the large vessels^31–33^.

#### Distance variation

During this scenario, the test subject is sitting in an office chair which is gradually moved away from the antenna so that the distance increases. It is attempted to keep the focus of the antenna on the same ROI during the distance variation. The test subject is moved in steps: the chair is moved away for around 5 … 10 cm and than kept still for around 15 … 20 s. This is repeated until the measurement time of 60 s is over. Using this scenario, it is attempted to determine the influence of the distance on the signal quality.

#### Speech

The speech scenario is aimed to observe the measurability of vital signs, in particular heart sounds, when the test subject is speaking during the measurement. A defined random text is given to the participants and is read aloud at an arbitrary speed until the measurement time is over.

#### Back

During this scenario, the ROI is located on the back. An ROI on the left side is chosen which is approximately at heart level. This way, the signal quality shall be maximized.

#### Angle variation

During angle variation, the angle between the thorax surface and the direction of the antenna is changed from a perpendicular 90° to both 60° and 120°.

### Subscenarios

In addition to the above mentioned scenarios, subscenarios are performed. These are performed in addition to the main scenarios, e.g., the post-exercise scenario in addition to a back measurement. Not all possible combinations are performed since this would result in an excessively large number of recordings.

#### Apnea

During apnea, the test subject is asked to breath freely during the first 30 s of the measurement and to hold the breath as long as possible during the second 30 s. It is furthermore distinguished if this maneuver is performed after inhalation or exhalation.

#### Post-exercise

These measurements are recorded after the test subject has done 20 squats. Through this stimulation of the cardiovascular system, heart rate, and cardiac output are increased.

Exemplary signals of three different scenarios can be seen in Fig. 2. Figure 2a shows a default measurement. Displayed are the raw radar distance signal, the filtered respiration sensor signal (0.05 Hz … 1.7 Hz), the three ECG leads (L1–L3), the radar signal filtered in the pulse frequency range (0.7 Hz … 15 Hz), the PCG signal, and the radar signal filtered in the heart sound frequency range (16 Hz … 80 Hz). For visibility reasons, only a 30 s segment of the whole measurement is shown. Figure 2b shows a distance variation measurement. As can be seen in the radar raw signal, the distance between antenna and body surface has been successively increased. After each increment, the person sat still again for a defined period of time. Please note that the radar distance signal is inverted for a better comparability with the respiration sensor signal. A movement towards the antenna is now reflected by a positive or rising radar signal while a negative signal indicates a target which is moving away. Figure 2c shows an apnea measurement. While the person was breathing normally in the beginning, the breath was hold from second eight and onwards.

## Data Records

The measurements were recorded using *Math Works MATLAB* and therefore stored as .mat files. All datasets are available online at *figshare*^34^ (10.6084/m9.figshare.c.4633958.v1). Furthermore, overview_and_rating.xlsx is available from figshare in which all recordings are listed to give an overview of the whole database. Both in the overview file as well as in the database itself the recordings are primarily split by person. Each folder in the database corresponds to one person. Next, the recordings of one person are separated by the measurement position of the PCG and the radar system (e.g., front or back) and further by the exact position of the systems (e.g., 2 L or 4 L). Within these folders, the data are separated by scenario or subtypes if applicable.

In the overview file, the recordings of each person are described in a separate sheet. Noted there are the exact times of the recordings, which also serve as unique file identifiers in the file names, subjective ratings of the signal qualities of the different sensor signals of a recording, and the exact positions and scenarios of the measurements.

The measurement position gives an exact indication about where both the PCG and the focus of the radar were placed on. The scenario describes the setting under which a single measurement was recorded. Table 2 shows the distribution of the scenarios in the database. For each scenario and each person, the number of recordings as well as the total length in seconds is displayed. Since the main purpose of the database was to prove the measurability of heart sounds using radar, the “Default” scenario is the most frequently appearing one. In total, the database consists of 13 376 s, or roughly 223 min, of synchronous recordings of all sensors. As mentioned in the Section “Procedures”, each measurement had an initial length of 60 s. However, due to cutting of the synchronisation sequence as well as rarely cutting away sequences with major external interferences (such as people walking into the room), the average length of one dataset is a bit less than 60 s.

**Table 2:** Overview of all test subjects showing the number of recordings per scenario per subject and the duration in seconds in parentheses.

PUT^a^	Default^b^	Carotid	Dist. Var.^c^	Speech	Back	Angl. Var.^d^	Total
1	27 (953 s)	3 (149 s)	0 (0 s)	0 (0 s)	1 (42 s)	0 (0 s)	31 (1144 s)
2	30 (1543 s)	2 (104 s)	1 (54 s)	0 (0 s)	0 (0 s)	2 (104 s)	35 (1805 s)
3	14 (735 s)	2 (72 s)	2 (88 s)	1 (55 s)	0 (0 s)	2 (107 s)	21 (1056 s)
4	13 (662 s)	3 (156 s)	0 (0 s)	0 (0 s)	1 (53 s)	0 (0 s)	17 (870 s)
5	8 (427 s)	4 (217 s)	2 (109 s)	1 (54 s)	2 (109 s)	3 (131 s)	20 (1047 s)
6	12 (615 s)	2 (109 s)	2 (109 s)	0 (0 s)	1 (53 s)	3 (161 s)	20 (1047 s)
7	24 (1302 s)	5 (270 s)	4 (164 s)	1 (49 s)	3 (163 s)	2 (107 s)	39 (2055 s)
8	12 (597 s)	3 (163 s)	1 (55 s)	0 (0 s)	0 (0 s)	3 (165 s)	19 (980 s)
9	17 (863 s)	3 (164 s)	1 (55 s)	2 (109 s)	1 (53 s)	3 (164 s)	27 (1409 s)
10	12 (660 s)	2 (111 s)	2 (101 s)	1 (55 s)	2 (110 s)	3 (164 s)	22 (1201 s)
11	7 (382 s)	2 (109 s)	1 (54 s)	0 (0 s)	1 (54 s)	3 (162 s)	14 (761 s)
Total	176 (8739 s)	31 (1625 s)	16 (789 s)	6 (322 s)	12 (637 s)	24 (1265 s)	265 (13376 s)

^a^Person-under-test. ^b^Radar in frontal position. ^c^Distance variation. ^d^Angle variation (±30°).

The ratings of the signal qualities comprise a rating of both the first and second heart sound of radar and PCG each, the ECG signal, and the signal of the respiration sensor. If a signal exhibits a high signal quality and more than 90% of the recording shows a clearly visible signal, it is labeled as “A”. A medium quality signal is labeled as “B” whereas a recording with low signal quality is labeled “C”. C-labeled signals mostly contain noise and do not show any characteristic features of that specific signal.

Table 3 summarizes the subjective ratings from the overview file. The table displays the number of ratings for each scenario and for each person, independent of the scenario. In this case, radar and PCG heart sounds (S1 and S2) are combined as a single rating. “A” is only assigned if both S1 and S2 are rated as “A”. “B” is assigned if one is “B” and the other is at least “B”. Measurements in which the ECG was not properly attached and no ECG signal of sufficiently high quality could be recorded were omitted since the ECG serves as main reference signal. Therefore, no ECG recording was labeled as C.

**Table 3:** Subjective quality rating of all measurements from all sensor signals.

PUT^a^	ECG			Breathing			Radar HS^b^			PCG HS^c^		
	A	B	C	A	B	C	A	B	C	A	B	C
1	20	11	0	10	6	15	15	12	4	19	7	5
2	30	5	0	11	4	20	27	6	2	29	3	3
3	13	8	0	4	3	14	17	2	2	15	4	2
4	15	2	0	8	5	4	2	10	5	7	7	3
5	19	1	0	16	0	4	3	8	9	7	9	4
6	17	3	0	17	0	3	7	5	8	14	6	0
7	25	14	0	29	2	8	22	14	3	19	7	13
8	19	0	0	18	0	1	9	8	2	9	3	7
9	26	1	0	19	2	6	14	8	5	7	6	14
10	22	0	0	18	2	2	17	4	1	20	2	0
11	14	0	0	14	0	0	8	3	3	11	3	0
Total	220	45	0	164	24	77	141	80	44	157	57	51

“A”, “B”, and “C” correspond to a high, a medium, and a low signal quality, respectively. Each number represents the number of recordings of a certain subject which falls into each rating.

^a^Person-under-test. ^b^Radar heart sound signal. ^c^PCG heart sound signal.

As mentioned above, the database consists of .mat files which each represent a single measurement. Each .mat file consists of several signals. ecg_lead3 and ecg_lead2 represent the ECG leads 3 (LA-LL) and 2 (RA-LL), respectively. respiration represents the raw signal of the respiration sensors. radar_I and radar_Q are the *I* and *Q* signal of the radar system. All signals are stored in. Furthermore, the .mat file contains the already synchronised PCG signal (pcg_audio) as well as the sampling rate Fs and a measurement information measurement_info which consists of the exact time of the recording and the number of the test subject. For each.mat file, a .csv file with the same name is located in the according folder. The .csv file contains the reference locations of both the R-peaks and the T-wave ends of the ECG signal. The labeling is done on all ECG leads using both the Pan-Tompkins QRS detection algorithm^35^ and the algorithm from Zhang *et al*.^36^ for locating the T-wave ends. The locations of the ECG lead with the highest signal quality were chosen as a first reference. Afterwards, all detected locations of this lead were manually inspected and corrected if needed. In case the labeling algorithms missed to detect R-peaks or T-wave ends, they were added manually. The first column of the .csv file represents the locations of the R-peaks and the second column represents the locations of the T-wave ends.

Both the subjective quality rating and the labeling of the ECG signals were done by two biomedical engineers with experience in the analysis of biomedical signals. Furthermore, a trained physician supported and revised the labeling.

## Technical Validation

**Fig. 3:**
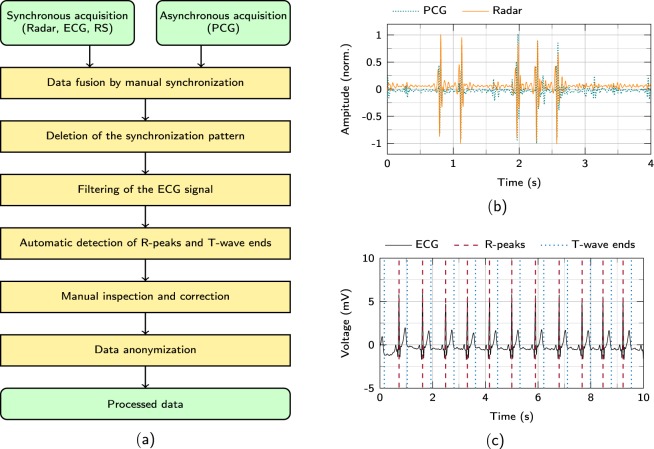
Synchronisation and reference labeling. (**a**) A flowchart showing the signal acquisition, synchronisation and ECG reference labeling process. (**b**) The tapping pattern used at the beginning of every measurement that is used to synchronise radar and PCG signal. (**c**) An exemplary reference labeling of an ECG signal showing the R-peaks and the T-wave ends^17^.

Figure 3a shows the overall flowchart of the data acquisition process. After each recording, the synchronously acquired radar, ECG and respiration sensor signals as well the PCG signal are acquired. In a first step, these signals are combined using a manual synchronisation. Afterwards, the synchronisation pattern is deleted. Next, the ECG signals are filtered to remove noise and in order to use algorithms for an automated detection of the R-peaks and T-wave ends. As mentioned above, these results are manually inspected and corrected if needed. As a last step, the recordings are anonymized and stored.

### Synchronisation between radar and PCG

After recording the raw signals, the PCG signal needs to be synchronised to the radar, ECG, and respiration sensor signal. Therefore, the test subject is tapped on the shoulder in a certain pattern at the beginning of each measurement. This pattern is visible both in the PCG and radar signal. To synchronise these signals afterwards, the signals are shifted until the patterns match as shown in Fig. 3b. The pattern is then removed from the recording.

### Correlation of radar and PCG heart sound signals

**Fig. 4:**
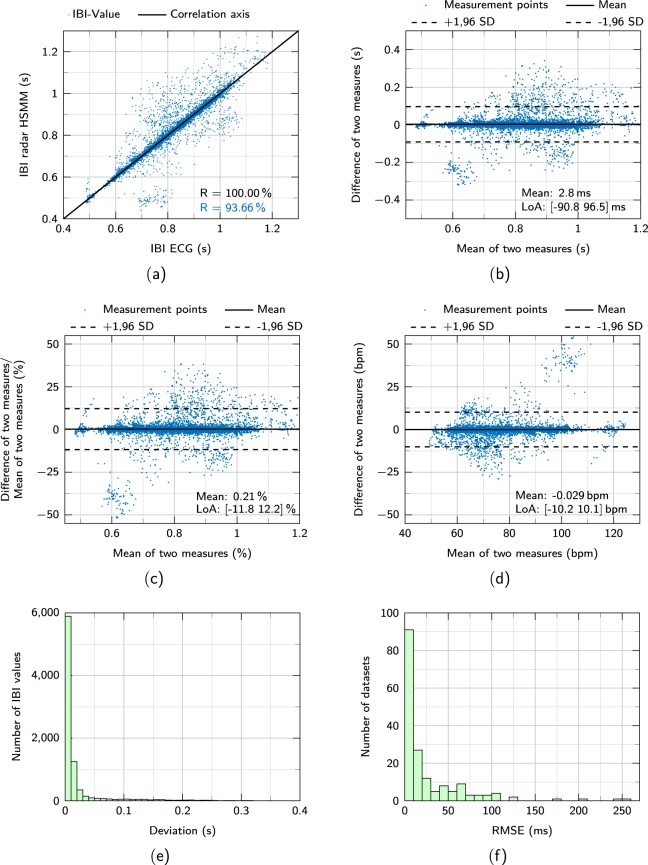
Performance comparison between radar and ECG. Scatter diagram (**a**) and Bland-Altman (**b**) plot of all IBI values. (**c**) shows the same Bland-Altman plot but with the differences plotted as percentage of the mean values. (**d**) shows the Bland-Altman plot with bpm values. (**e**) Histogram showing the deviation of all IBI values from their reference. (**f**) Number of datasets that have a certain RMSE.

To illustrate the relationship between the reference ECG signals and the radar-recorded heart sound signals, the two quantities are correlated with each other. First, the interbeat-intervals (IBIs) between successive heartbeats are calculated. For the ECG, the R-peaks are defined as points in time where a heartbeat occurs. In case of the radar heart sounds, the HSMM algorithm^37^ is used to segment the signal into the four states “first heart sound (S1)”, “systole”, “second heart sound (S2)”, and “diastole”. The start of each S1 is defined as a single heartbeat. Using both the R-peaks and the starts of S1, the IBI values can be calculated for each signal. These IBI signals are both filtered by a median filter of size five and a moving average filter of size six to compensate for artifacts. In order to correlate the IBI values, values at equidistant and identical points in time are necessary. For this purpose, the IBI signals are resampled to the sampling frequency of 2000 Hz and a IBI value is taken at every second. These IBI values are then correlated and plotted in Fig. 4a. Please note that only recordings from the default scenario are chosen for the correlation. The solid black line depicts a perfect correlation of 100%. Low scattering and a high Pearson correlation coefficient of $R=93.66 \%$ can be observed. This high correlation value as well as the large number of samples indicate that the radar measures highly similar heart rate values compared to the ECG. Although the ECG is considered as reference device, it also contains some amount of noise and the labeling might introduce some error. Therefore, a correlation of 100% is practically impossible to achieve.

However, a high correlation value and a correlation plot might not necessarily lead to a good agreement between two measuring methods^38^. This is why Bland and Altman introduced the so-called Bland-Altman plot^39^. IT has since been used to compare different measuring methods or compare new methods to gold standard techniques. The plot allows to identify any systematic errors such as a fixed or proportional bias^38^. A fixed bias indicates that there is a constant offset between the two measuring methods whereas a proportional bias means that the difference between the two measuring methods depends on the absolute value. These differences would usually not be visible when performing correlation analysis. Figure 4b shows the Bland-Altman plot of the same IBI values that are seen in Fig. 4a. Again, the IBIs are used as they resemble the instantaneous heart rate. The Bland-Altman analysis plots the differences of the IBI values of the two measuring methods against the corresponding mean values. The mean difference between the two methods is indicated by a solid black line while the two dashed black lines represent the lower and upper limits of agreement (LoA). They are calculated by adding and subtracting 1.96 times the standard deviation (SD) of the differences to the mean. A very low fixed bias of 2.8 ms is observed which indicates that there is no constant offset between the two methods. Furthermore, there is no increasing or decreasing trend over the entire interval, which shows that no proportional bias is influencing the measurements. The LoAs are at −90.8 ms and 96.5 ms. These values imply a low deviation between the two methods. Figure 4c shows the Bland-Altman plot of the IBI values but with the differences plotted as percentage of the mean value. Again, a small bias of 0.21% is observed with the LoAs at −11.8% and 12.2%. No proportional bias can be noticed. In Fig. 4d, the Bland-Altman plot for the beats-per-minutes (bpm) values is displayed. The bpm values are simply calculated by dividing 60 through the IBI values. Low IBI values therefore correspond to a high bpm value and vice versa. The mean bias in this case is −0.029 bpm with the LoAs being at −10.2 bpm and 10.1 bpm. Again, no proportional trend is observed.

The histogram in Fig. 4e shows the deviation of all single IBI values from their reference values with a bin width of 10 ms. Overall, 8554 datapoints are evaluated. Almost 6000 of these IBI values have a deviation of less than 10 ms, which corresponds to 68.90% of all points. 90% of all IBI values have a deviation of less than 45.0 ms. These results indicate a high similarity between the radar and the ECG IBI values. The histogram in Fig. 4f shows the number of datasets that have a certain root-mean-square error (RMSE) of the IBI values. For this purpose, the RMSE value of each single dataset is calculated, whereby one dataset represents one measurement. The average RMSE is 47.85 ms while more than 90 of the 176 datasets have an RMSE of less than 10 ms.

### Detailed analysis

Next, some specific scenarios besides the default scenario shall be discussed in detail. This includes the effects of the measuring position, the measurability in the back area, and the robustness of the radar system towards speech artifacts.

#### Effects of the measuring position

The measuring position denotes the alignment of the antenna, i.e., the position on the body surface on which the antenna is focused on. The different positions can be seen in Fig. 1a. In general, all positions allow for a good measurability of the heart sounds. However, the measurability at a certain position varies from person to person. This is due to the fact that the exact anatomy of every person is a little different such as the exact location of the heart inside the chest. Other factors, such as different fat distribution, also play a role. Since the radar integrates a relatively large area, the exact alignment of the antenna does not play an essential role. In principle, the amplitude of S1 is larger than the amplitude of S2 since the origin of the first heart sound (tension of the ventricle muscles) is stronger than the source of the second heart sound^30,40,41^.

**Fig. 5:**
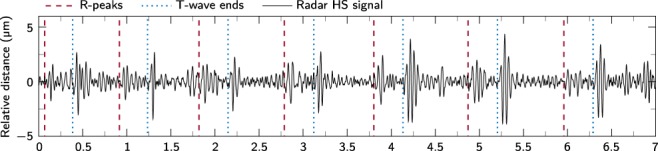
Effects on the signal dynamics depending on the measuring position. Radar heart sound signal of test subject 7 with the antenna being focused on 2R. The S2 have a higher amplitude compared to the S1.

However, there are measuring positions where the amplitude of S2 can exceed that of S1. On the one hand, this is the case with measurements at the carotid arteries, as they are much closer to the aortic and pulmonary valves, where the S2 originates, than to the ventricle muscles and the tip of the heart. Furthermore, if the heart sounds are recorded directly above the aortic and pulmonary valves, the amplitude of S2 may exceed that of S1. Figure 5 shows a measurement of test subject 7 with the antenna focused on 2R. In this area the aortic and pulmonary valves are expected. As can be seen, the amplitude of the second heart sounds is larger than that of the first heart sounds. Overall, it can be observed that the heart sounds on the left frontal half of the upper body are better perceptible than on the right half. This is also related to the human anatomy, as the heart is mostly located on the left side of the chest^30^.

#### Measurability in the back area

**Fig. 6:**
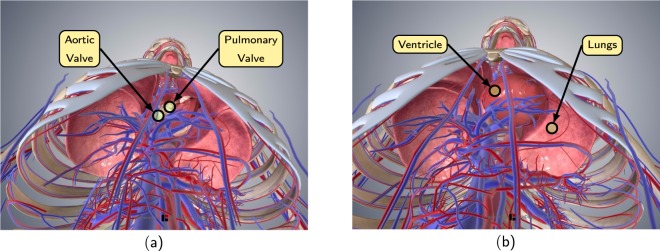
Location of the heart, the lung, and the aortic and pulmonary valves. In general, the heart has a ventral position in the thorax^30^. The images are taken from Biodigital Inc. (https://human.biodigital.com/index.html).

Measurements in the back area showed a large interindividual difference. In some subjects, the measurability of the heart sounds in the back area is so poor that no useful data can be recorded either with the stethoscope nor with the radar. Other test subjects however had a medium or good measurability of the heart sounds in the back area. Furthermore, while the first heart sound is clearly perceptible at some times, the second heart sound is consistently difficult to detect in the back region. This could be due to the location of the heart, especially the aortic and pulmonary valves. Figure 6 illustrates this. As can be seen, the ventricles are located quite centrally in the upper body, the aortic and pulmonary valves however are positioned rather frontally. In addition, the lung is located between the heart and the back. The dampening by the lung in addition to the fact that S2 is generally harder to measure compared to S1 explains the low signal quality of the second heart sound in the back area.

#### Robustness against speech artifacts

**Fig. 7:**
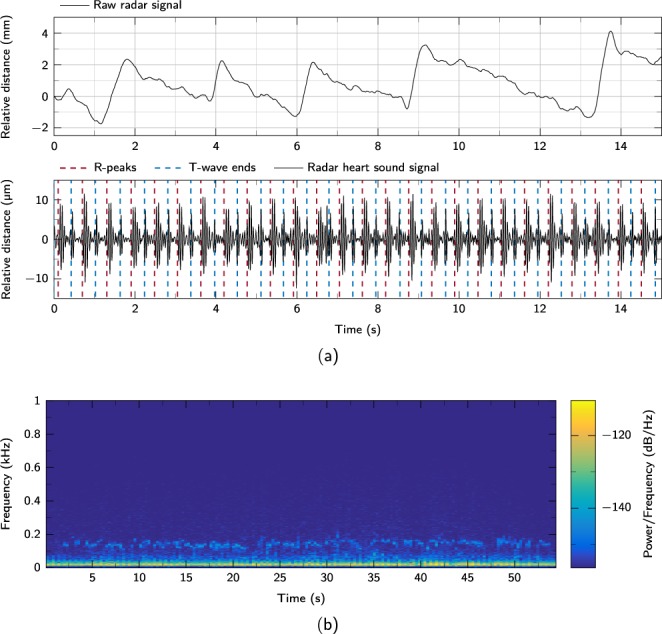
Effects of speech on the measurement. (**a**) A cutout of a raw distance signal and the corresponding radar heart sound signal of test subject 3 while reading a text. (**b**) Short-Time Fourier Transform of the radar signal in (**a**) bandpass-filtered with a passband of 16 Hz and 1000 Hz.

In this scenario, a text is read by the test subjects while the vital signs are recorded. Figure 7 shows the section of a measurement from subject 3 while reading the text aloud. The typical breathing curve resulting from reading is clearly visible in the raw signal. The rapid increase indicates the moments at which the subject breathes in during a short speech break. Afterwards, the subject breathes out continuously during the speech. In the filtered signal the heart sounds can be easily recognized. Figure 7b shows the short-term FFT (STFT) of the measurement in Fig. 7a. The radar signal is filtered between 16 Hz and 1000 Hz. The increased upper cutoff frequency of 1000 Hz enables the detection of speech components in the signal. A higher upper limit is not possible due to the sampling rate of 2000 Hz. The heartbeats in the range below 100 Hz are clearly visible. The speech artifacts are clearly recognizable in the range between 100 Hz and 200 Hz. Since there is no overlap, both components can be separated without problems.

## Usage Notes

The whole dataset is freely available at *figshare*. All the records are stored in .mat format and can be analyzed in *MATLAB*. These files can of course also be converted into different formats to use with other software applications.

As described in the Section “RF front end and Six-Port radar”, the distance signal can easily be reconstructed from *I* (radar_I) and *Q* (radar_Q) using a simple arctangent demodulation. However, due to nonidealities at the front end, amplitude, and phase imbalances occur inside the Six-Port structure. This leads to offset, gain and phase errors within the sampled signals. Before the demodulation can be applied, an ellipse fitting algorithm such as presented in^42^ has to be employed. Further descriptions can be taken from^17^. An example is also given in the code samples which are available online.

## Data Availability

All custom user code used for the technical validation is available from https://gitlab.com/kilinshi/scidata_vsmdb. Furthermore, a dataset viewer is also included in the repository which can be used to easily view any dataset that is loaded into the *MATLAB* workspace. The code was written and tested using *MATLAB* R2018b for *Microsoft Windows*.

## References

[1] Balaji S, Ellenby M, McNames J, Goldstein B (2002). Update on intensive care ECG and cardiac event monitoring. Card. Electrophysiol. Rev..

[2] Caples SM, Hubmayr RD (2003). Respiratory monitoring tools in the intensive care unit. Curr. Opin. Crit. Care..

[3] Banerjee A, Girard TD, Pandharipande P (2011). The complex interplay between delirium, sedation, and early mobility during critical illness: applications in the trauma unit. Curr. Opin. Anesthesiol..

[4] Stiller K (2007). Safety issues that should be considered when mobilizing critically ill patients. Crit. Care Clin..

[5] Cohen IL (2002). Management of the agitated intensive care unit patient. Crit. Care Med..

[6] Sowan, A. K., Tarriela, A. F., Gomez, T. M., Reed, C. C. & Rapp, K. M. Nurses’ perceptions and practices toward clinical alarms in a transplant cardiac intensive care unit: Exploring key issues leading to alarm fatigue. *JMIR Hum*. *Factors* **2** (2015). 10.2196/humanfactors.4196 PMC4797660 27025940

[7] Kim, C.-S. *et al*. Ballistocardiogram: Mechanism and potential for unobtrusive cardiovascular health monitoring. *Sci*. *Rep*. **6** (2016). 10.1038/srep31297 PMC4977514 27503664

[8] Gubner RS, Rodstein M, Ungerleider HE (1953). Ballistocardiography. Circulation.

[9] Shafiq G, Veluvolu KC (2014). Surface chest motion decomposition for cardiovascular monitoring. Sci. Rep..

[10] Wang CC (2007). Human life signs detection using high-sensitivity pulsed laser vibrometer. IEEE Sens. J..

[11] Scalise, L. Non contact heart monitoring. In *Advances in Electrocardiograms-Methods and Analysis* (InTech, 2012).

[12] Morbiducci U, Scalise L, De Melis M, Grigioni M (2007). Optical vibrocardiography: A novel tool for the optical monitoring of cardiac activity. Ann. Biomed. Eng..

[13] Aardal Ø (2013). Physical working principles of medical radar. IEEE Trans. Biomed. Eng..

[14] Li C, Lubecke VM, Boric-Lubecke O, Lin J (2013). A review on recent advances in Doppler radar sensors for noncontact healthcare monitoring. IEEE Trans. Microw. Theory Techn..

[15] Li C (2017). A review on recent progress of portable short-range noncontact microwave radar systems. IEEE Trans. Microw. Theory Techn..

[16] Sakamoto T (2016). Feature-based correlation and topological similarity for interbeat interval estimation using ultrawideband radar. IEEE Trans. Biomed. Eng..

[17] Will C (2018). Radar-based heart sound detection. Sci. Rep..

[18] Will C (2017). Local pulse wave detection using continuous wave radar systems. IEEE J-ERM.

[19] Pisa S, Pittella E, Piuzzi E (2016). A survey of radar systems for medical applications. IEEE Aero. El. Sys, Mag..

[20] Zhu F, Wang K, Wu K (2018). A fundamental-and-harmonic dual-frequency doppler radar system for vital signs detection enabling radar movement self-cancellation. IEEE Trans. Microw. Theory Techn..

[21] Xiong Y, Chen S, Dong X, Peng Z, Zhang W (2017). Accurate measurement in doppler radar vital sign detection based on parameterized demodulation. IEEE Trans. Microw. Theory Techn..

[22] Ren L (2017). Comparison study of noncontact vital signs detection using a doppler stepped-frequency continuous-wave radar and camera-based imaging photoplethysmography. IEEE Trans. Microw. Theory Techn..

[23] Mercuri M (2019). Vital-sign monitoring and spatial tracking of multiple people using a contactless radar-based sensor. Nat. Electron..

[24] Lee Y (2018). A novel non-contact heart rate monitor using impulse-radio ultra-wideband (ir-uwb) radar technology. Sci. Rep..

[25] Liang X, Deng J, Zhang H, Gulliver TA (2018). Ultra-wideband impulse radar through-wall detection of vital signs. Sci. Rep..

[26] Park J-Y (2019). Preclinical evaluation of a noncontact simultaneous monitoring method for respiration and carotid pulsation using impulse-radio ultra-wideband radar. Sci. Rep..

[27] Leatham A (1958). Auscultation of the heart. Lancet.

[28] Wells B (1954). The assessment of mitral stenosis by phonocardiography. Br. Heart J..

[29] Koelpin A (2016). Six-Port based interferometry for precise radar and sensing applications. Sensors.

[30] Hall, J. E. *Textbook of Medical Physiology* (Elsevier Health Sciences, 2015).

[31] Levick, J. R. *An introduction to cardiovascular physiology* (Butterworth-Heinemann, 2013).

[32] Farber JJ, Purvis JH (1963). Conduction of cardiovascular sound along arteries. Circ. Res..

[33] Smith D, Ishimitsu T, Craige E (1984). Mechanical vibration transmission characteristics of the left ventricle: implications with regard to auscultation and phonocardiography. J. Am. Coll. Cardiol..

[34] Shi K (2019). figshare.

[35] Pan J, Tompkins WJ (1985). A real-time QRS detection algorithm. IEEE Trans. Biomed. Eng..

[36] Zhang Q, Manriquez AI, Medigue C, Papelier Y, Sorine M (2006). An algorithm for robust and efficient location of t-wave ends in electrocardiograms. IEEE Trans. Biomed. Eng..

[37] Springer DB, Tarassenko L, Clifford GD (2016). Logistic regression-HSMM-based heart sound segmentation. IEEE Trans. Biomed. Eng..

[38] Giavarina D (2015). Understanding bland altman analysis. Biochem. Medica.

[39] Bland JM, Altman D (1986). Statistical methods for assessing agreement between two methods of clinical measurement. Lancet.

[40] Levine SA, Harvey WP (1960). Clinical auscultation of the heart. Acad. Med..

[41] Braunwald, E., Zipes, D. P. & Libby, P. *Heart disease: A textbook of cardiovascular medicine*, 6th edn. (WB Saunders, 2001).

[42] Singh A (2013). Data-based quadrature imbalance compensation for a CW doppler radar system. IEEE Trans. Microw. Theory Techn..

